# The Status of the Acupuncture Mechanism Study Based on PET/PET-CT Technique: Design and Quality Control

**DOI:** 10.1155/2019/9062924

**Published:** 2019-11-29

**Authors:** Zhaoxuan He, Likai Hou, Ruirui Sun, Tao Yin, Peihong Ma, Li Chen, Shirui Cheng, Xiaoyan Liu, Xiaojuan Hong, Jian Hou, Fang Zeng

**Affiliations:** ^1^Acupuncture and Tuina School/The 3rd Teaching Hospital, Chengdu University of Traditional Chinese Medicine, Chengdu, Sichuan, China; ^2^Acupuncture & Brain Science Research Center, Chengdu University of Traditional Chinese Medicine, Chengdu, Sichuan, China; ^3^Clinical Medicine College/Teaching Hospital, Chengdu University of Traditional Chinese Medicine, Chengdu, Sichuan, China

## Abstract

PET/PET-CT is an important technique to investigate the central mechanism of acupuncture *in vivo*. This article collected original research papers with keywords of “Acupuncture,” “PET,” “PET/CT,” and “Positron emission tomography” in PubMed and CNKI databases from January 2003 to December 2018. As a result, a total of 43 articles were included. Based on the literature analyses, we found that (1) reasonable arrangement of the operation process and the choice of appropriate acupuncture intervention time is conducive to a better interpretation of acupuncture-PET/PET-CT mechanism and (2) the selection of participants, sample size, acupuncture intervention, and experimental conditions would affect study results. Therefore, effective quality control is an important way to ensure the repeatability of research results.

## 1. Introduction

Acupuncture, as an essential component of traditional Chinese medicine (TCM), is increasingly accepted as an alternative and complementary therapy by western countries for its reliable effect of relieving pain and regulating visceral functions [1–3]. Since the 1970s, a number of studies have confirmed that the central nervous system (CNS) plays an important role in acupuncture effect [4, 5]. With the development of neuroimaging techniques, using functional Magnetic Resonance Imaging (fMRI) and Positron Emission Tomography (PET) to investigate the cerebral responses to acupuncture stimulation *in vivo* has gradually become a spotlight in acupuncture mechanism research. After analyzing the acupuncture-neuroimaging articles published from 1995 to 2014, we found that MRI and PET/PET-CT are the two imaging technologies most commonly used [6].

PET, as the representative of biochemical imaging, becomes an important technique to explore the acupuncture mechanism for its higher functional resolution and significant integrity [7, 8]. Combining the functional metabolic imaging and anatomical imaging, positron emission tomography-computed tomography (PET-CT) stands for the highest level of current nuclear medical imaging techniques. It can clearly reflect the change of corresponding functional areas of the brain after stimulus, so PET/PET-CT has been widely used in brain research studies [9]. In the past two decades, many investigators had mapped the central activity changes elicited by acupuncture stimulation using PET/PET-CT which provided visualized and reliable interpretation for acupuncture treating stroke, depression, and other diseases [10–12].

However, the study designs, especially the scanning procedures of these acupuncture-PET/PET-CT studies, have significant differences. To our knowledge, there are at least 6 kinds of scanning procedures used in the acupuncture study. The methodological differences limit the further application of PET/PET-CT in acupuncture studies and affect the repeatability and reliability of the results of these studies.

So, this study aims to analyze the status of the acupuncture-PET/PET-CT studies from the study design and the quality control by comparing the original articles from 2003 to 2018, so as to provide references for future studies.

## 2. Methods

### 2.1. Searching Strategy

We searched China National Knowledge Infrastructure (CNKI, 1979–2018) for articles from January 1, 2003, to December 31, 2018, in Chinese language by the title/abstract search. The detailed search terms and search strategies are as follows: (“Acupuncture” (*Zhenjiu*) OR “Needling” (*Zhenci*) OR “Manual acupuncture” (*Shouzhen*) OR “Electroacupuncture” (*Dianzhen*) OR “Scalp acupuncture” (*Touzhen*)) AND (“PET” OR “PET/CT” OR “Positron emission tomography” OR “Positron emission tomography with computed tomography”). Also, we searched the original articles published from January 1, 2003, to December 31, 2018, in PubMed (1959–2018) using the following Mesh terms and search strategies:(“Acupuncture”[Mesh terms] OR “Acupuncture Therapy”[Mesh terms] OR “Acupuncture, Ear” OR “Acupuncture Points”[Mesh terms] OR “Acupuncture Analgesia”[Mesh terms]) AND (“Positron emission tomography”[Mesh terms]).

We screened the bibliographies of identified trials and reviewed articles for further potentially relevant publication. Subsequently, we screened the full texts and assessed whether these articles met the inclusion criteria.

### 2.2. Inclusion and Exclusion Criteria

The articles would be included, if they were: (1) original articles; (2) acupuncture-PET/PET-CT study on human beings; (3) published in English or Chinese; and (4) published from January 1, 2003, to December 31, 2018.

**Figure 1:**
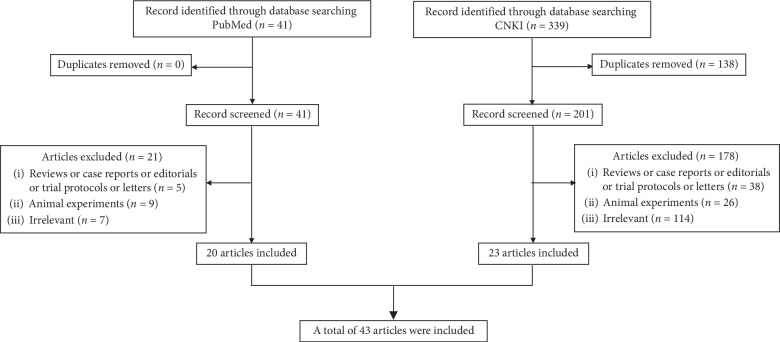
The flowchart of literature search and screening process.

The articles would be excluded, if they were: (1) reviews, case reports, editorials, trial protocols, or letters; (2) acupuncture-PET/PET-CT study on animals; or (3) duplicate articles. (Figure 1 shows the flowchart of the literature selection).

### 2.3. Data Extraction and Analysis

We extracted the data including characteristics of the participants (patients or the health, accompanying symptoms), sample size, acupuncture intervention (method of intervention, manipulation procedure, *Deqi*/needle sensation, and acupuncturist), PET/PET-CT scanning process, and imaging agent types. Data analysis was conducted after data extraction.

## 3. Results

Forty-three original articles were included.

### 3.1. Annual Distribution of the Studies

**Figure 2:**
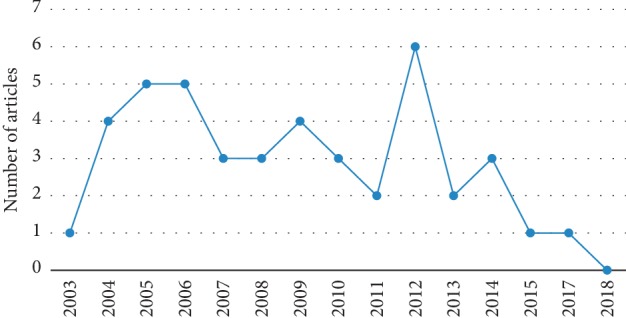
Annual distribution of the acupuncture-PET/PET-CT studies.

The annual distribution of the studies was shown in Figure 2. In 2012, the acupuncture studies using PET/PET-CT reached the peak.

### 3.2. Classification of Participants

**Figure 3:**
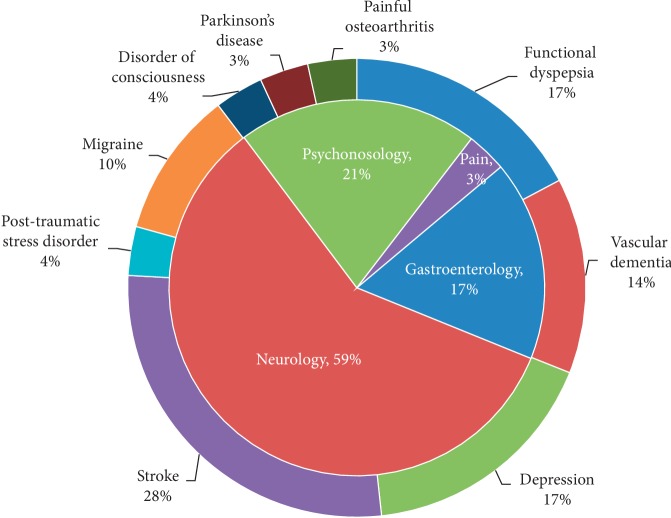
The distribution of disease system and disease category involved in acupuncture-PET/PET-CT studies.

Fourteen studies were performed on healthy subjects [13–26]. Twenty-three studies were performed on patients, involving 10 diseases in 4 systems [10–12, 27–46]. Six studies recruited both healthy subjects and patients [47–52]. (Figure 3).

### 3.3. Sample Size

The average sample size of these studies was 8 participants per group. There is no difference in sample size between the studies performed on healthy subjects and patients.

### 3.4. Acupuncture Intervention

#### 3.4.1. Acupuncture Modalities

Nineteen studies chose manual acupuncture as the intervention method [10, 13, 20–25, 28, 29, 32, 33, 35–37, 39, 43, 46, 47]. Twenty-four studies chose electroacupuncture as the intervention method [11, 12, 14–19, 26, 27, 30, 31, 34, 38, 40–42, 44, 45, 48–52].

#### 3.4.2. Manipulation Procedure

Except 1 paper which did not describe the acupuncture manipulation [32], the other 42 articles described the manipulation procedure of acupuncture.

#### 3.4.3. Deqi (Needle Sensation)

Thirty-three studies emphasized the needle sensation (*Deqi*) during acupuncture stimulation [11, 13–20, 22, 23, 26–31, 34, 36–42, 44, 46–52].

Two studies evaluated the needle sensation with the 10-point Visual Analogue Scale (VAS) and the Needle Sensation Questionnaire (NSQ) [22, 46].

#### 3.4.4. Qualification of Acupuncturists

Fourteen articles mentioned the qualification of acupuncturists [13, 15, 16, 20, 21, 23, 24, 27, 30, 33, 39, 41, 43, 47].

### 3.5. PET/PET-CT Scanning Process

**Figure 4:**
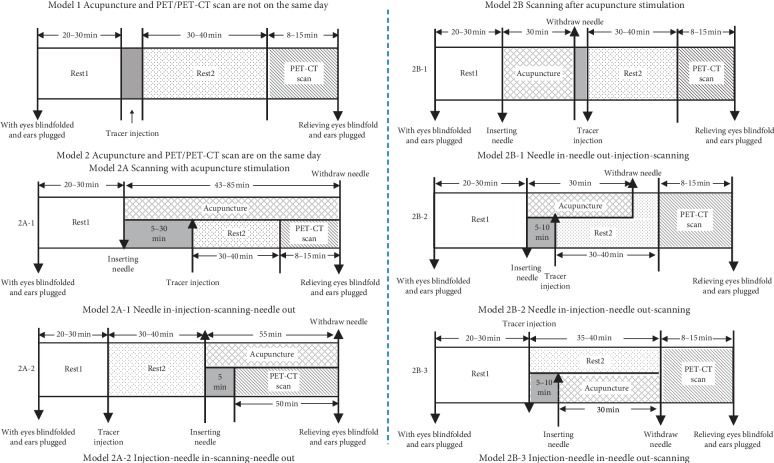
The models of PET/PET-CT scan.

The PET/PET-CT scanning processes can be divided into two categories: (1) acupuncture and PET/PET-CT scan are not on the same day (Figure 4, Model (1) [11, 12, 28, 29, 32, 34, 35, 37, 39, 42, 44–46, 48, 51] And (2) acupuncture and PET/PET-CT scan are on the same day (Figure 4, Model (2) [10, 13–27, 30, 31, 33, 36, 38, 40, 41, 43, 47, 49, 50, 52].

According to the timing of acupuncture stimulation in the scanning process, the Model 2 can be divided into two subtypes: scanning with acupuncture stimulation (Figure 4, Model 2A) [15–17, 25, 26, 43, 49, 50, 52] and scanning after acupuncture stimulation (Figure 4, Model 2B) [10, 13, 14, 18–24, 27, 30, 31, 33, 36, 38, 40, 41, 47]. According to the sequence of acupuncture stimulation and tracer injection, the Model 2A can be divided into two subtypes: injecting after inserting needle (Figure 4, Model 2A-1) [25, 43, 49, 50, 52] and injecting before inserting needle (Figure 4, Model 2A-2) [15–17, 26]. The Model 2B can be divided into three subtypes: injecting during inserting needle (Figure 4, Model 2B-1) [14, 18, 30, 38], injecting after inserting needle (Figure 4, Model 2B-2) [10, 13, 19, 20, 22–24, 27, 31, 33, 36, 40], and injecting before inserting needle (Figure 4, Model 2B-3) [21, 41, 47].

**Figure 5:**
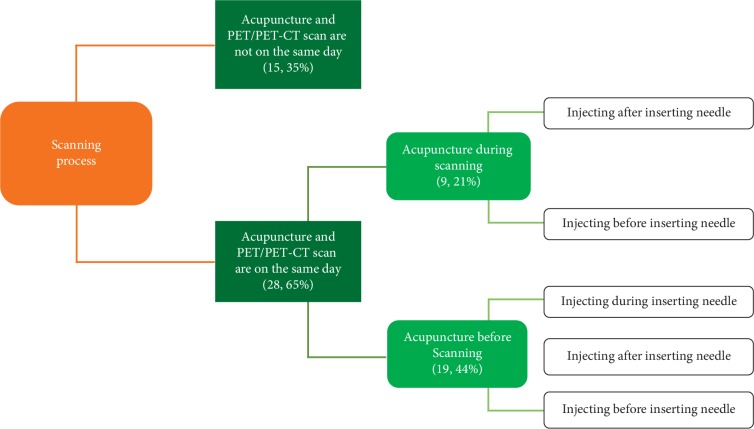
PET/PET-CT scanning process classification.

Studies of Model 2 were the largest proportion (65%), and studies of Model 1 accounted for about 35% (Figure 5).

### 3.6. Imaging Agent Types

Among the imaging agents, fluorine-18 fluorodeoxyglucose(18F-FDG) (95%) was most commonly used in the PET/PET-CT scanning, and only two papers used labeled water (5%) [25, 46].

## 4. Discussion

As a main neuroimaging technique, PET/PET-CT shows an irreplaceable advantage in biochemical imaging and becomes an important method to explore the central mechanism of acupuncture. Using PET/PET-CT, investigators had mapped the central responses to acupuncture treatment not only on healthy subjects but also on patients with neuropathy, psychological disorders, gastrointestinal disorders, and pain (Figure 3).

In this study, we found that, from 2003 to 2012, the number of acupuncture-PET/PET-CT studies increased and peaked in 2012, but decreased significantly thereafter. The reasons for this decline might be due to its expensive cost, non-negligible radiation risk, and unregular experimental design.

With the advent of the hybrid PET/MR and its gradual application in clinic and research, people have begun to try to apply it in acupuncture research. So, it is necessary to review the methodological issues in the acupuncture-PET studies, so as to provide reference for optimizing the future study. Among these methodological issues involved in the study design, there are two main aspects in the disagreement in these studies. One is the process of PET/PET-CT scan, and the other is the quality control-related information.

### 4.1. The Process of PET/PET-CT Scan

Owing to different experimental condition and purpose, the PET/PET-CT scanning process was quite different. There are at least six kinds of scan processes recorded in these papers. This difference is heightened in the timing of acupuncture stimulation.

#### 4.1.1. The Types of PET/PET-CT Scanning Process

In the study, the PET/PET-CT scanning processes were divided according to three aspects: (1) whether the acupuncture stimulation was performed on the same day as the PET/PET-CT scan. According to this, the scanning processes were divided into two models: Model 1, acupuncture and PET/PET-CT scan were not performed on the same day and Model 2, acupuncture and PET/PET-CT scan were performed on the same day. (2) Whether the scanning is performed simultaneously with acupuncture stimulation. According to this, the Model 2 was divided into two types: Model 2A is scanning with acupuncture stimulation, and Model 2B is scanning after acupuncture stimulation. (3) What is the timing of acupuncture stimulation and tracer injection? According to this, the Model 2A was divided into two subtypes (Model 2A-1 and Model 2A-2), and the Model 2B was divided into three subtypes (Model 2B-1, Model 2B-2, and Model 2B-3). (Figures 4 and 5).

#### 4.1.2. The Characteristics of Each Type

The main feature of Model 1 is to scan completely in a resting state. It follows the routine scan process including resting, tracer injection, resting, and then scanning (Figure 4). The advantage of this model is that there is less interference in the scanning process.

The main characteristic of Model 2 is that the scanning and acupuncture stimulations are performed on the same day.

For Model 2A, it is marked by scanning and acupuncture stimulation at the same time. Its advantage is that it can achieve the most significant cerebral responses to acupuncture stimulation. However, scanning with acupuncture needles retained in the body might bring some disturbing factors and risk factors. The Model 2A is mainly applied to investigate the central mechanism of the instant effect of acupuncture stimulation on the patients with paroxysmal diseases or healthy subjects.

The main differences between Model 2A-1 and Model 2A-2 are the timing of acupuncture needle insertion and tracer injection and the retaining time of the needles. Compared with Model 2A-2, Model 2A-1 shows longer retaining time of the needles. In some study, the retaining time is even more than 1 hour. It is not very consistent with clinical practice.

For Model 2B, it is characterized by scanning after acupuncture stimulation. Its advantage lies in not only gaining the obvious cerebral responses elicited by acupuncture but also avoiding the interference of acupuncture manipulation on scanning.

The main difference among these three subtypes of model 2B is also the time of acupuncture stimulation and tracer injection. In details, in Model 2B-1, the tracer injection is performed just after withdrawing the acupuncture needle (acupuncture stimulation is over). The order of acupuncture and tracer injection is needle in-needle out-tracer injection. In Model 2B-2, the tracer injection is performed during the period of acupuncture stimulation. The order of acupuncture and tracer injection is needle in-tracer injection-needle out. In Model 2B-3, the tracer injection is performed before inserting the acupuncture needle (acupuncture stimulation is begun). The order of acupuncture and tracer injection is tracer injection-needle in-needle out.

#### 4.1.3. The Reasons of These Different Designs

The reasons of these different designs can be analyzed from 3 aspects.

Firstly, whether the acupuncture stimulation and PET/PET-CT scan are performed on the same day is decided by the purpose of study. The Model 1 is applied to explore the mechanism of the long-term effect of acupuncture treatment, while the Model 2 is more suitable for investigating the instant effect of acupuncture.

Secondly, whether the scanning and acupuncture stimulation are performed at the same time depends on a comprehensive assessment of the participants' physical conditions, potential risk, and interference factor.

Thirdly, the issue of who is the first to inject the tracer and to insert needles reflects two considerations of investigators. One is the half-life of the tracer. For example, the half-life of 18F-FDG is 109.8 minutes. It is reported that after entering the body for 40 minutes, its metabolism tends to stabilize [53]. So, the PET/PET-CT scan should be performed after injection of 18F-FDG at 35–40 minutes. For the same reason, it is better to perform acupuncture stimulation after 35–40 minutes by injecting 18F-FDG. In this way, it can make the responsive process of acupuncture signal in the brain basically coincide with the process of 18F-FDG metabolism stabilizing in the brain. The other is whether the sharp pain induced by the tracer injection significantly influences cerebral activity. Some investigators hold that the sharp pain induced by the tracer injection is stronger than the needle sensation, and it can elicit significant cerebral activity changes and result in false positive results, so they selected inserting needles after tracer injection [54]. Compared to Model 2B-1 and Model 2B-2, Model 2B-3 takes these two aspects mentioned above into consideration.

### 4.2. The Quality Control

Due to the complexity of the brain function and the variety of acupuncture manipulations, the selection of participants, sample size, acupuncture intervention, and experimental condition would affect the results of the study. Therefore, seeking a more reasonable and practical method to improve the reproducibility and reliability of results seems to be important to acupuncture-neuroimaging study.

#### 4.2.1. The Selection of Participants

Based on the literature analyses, we found that the majority of PET/PET-CT studies (55%) were performed on patients. According to the theory of traditional Chinese acupuncture, acupuncture treatment acts on strengthening the body resistance to removing pathogenic factors, and the efficacy of acupuncture treatment is specific to the pathological state, not the physiological conditions [55]. The function of acupoint is body condition dependent, and the acupoint is more active during the pathological conditions than in a healthy state [56, 57]. So, patients should be a better choice for acupuncture-neuroimaging studies [6]. In this study, we also found that the diseases of the nervous system were the most common choice in the acupuncture-PET/PET-CT study. The result indicated that acupuncture has a good effect for diseases of the nervous system, such as stroke and depression [10–12]. However, acupuncture does better in regulating functional disorders rather than curing organic diseases [58]. More attention could be paid on the mechanism of acupuncture regulating functional disorders.

In addition, the subtypes of disease should be taken into consideration when you choose patients as the participants in the neuroimaging study. Patients with different subtypes might have cerebral functional or/and structural differences. In previous studies, we compared with epigastric pain syndrome (EPS), and acupuncture has a more significant effect in the treatment of FD in patients with postprandial distress syndrome (PDS) [59]. So, in acupuncture-neuroimaging study, the same subtype of a disease can be chosen to ensure the homogeneity of participants and reduce the baseline state of brain function of subjects.

#### 4.2.2. Sample Size

Due to the potential radioactivity and the costs of imaging, the sample size in most of PET/PET-CT studies was small. Most studies controlled sample size between 6 and 15 cases. In this study, we found that the average sample size was 8 participants per group, no matter the healthy subjects and patients. Some researchers hold that the activated brain regions are related to the sample size [60]. Nowadays, most investigators agreed that a bigger sample size brought the achievement of stable statistical power [61].

#### 4.2.3. Acupuncture Intervention

As we know, the defined influence of the qualification of the acupuncturist and manipulation procedure has an effect on clinical efficacy. In clinical studies, we should formulate the standard process of the acupuncture operation that included numbers of needle, depth of insertion, elicited response, and needle retention time. Practitioners should choose a qualified professional acupuncturist and the same acupuncturist in one research as far as possible.

*Deqi* (needle sensation) plays an important role in acupuncture efficacy. A majority of clinical trials have demonstrated that the curative effect with needle sensation was superior to those without needle sensation [62]. A neuroimaging study also showed a significant difference in cerebral responses under the *Deqi* and non-*Deqi* condition [63]. So, it is important to require *Deqi* in acupuncture-neuroimaging studies.

However, we found that the questionnaire-based forms were less used to assess the needle sensation in acupuncture-PET-CT studies. There were only two kinds of forms such as 10-point Visual Analogue Scale (VAS) and Needle Sensation Questionnaire (NSQ) applied to these researches.

#### 4.2.4. Experimental Condition

In brain imaging studies, the individual's psychological activity, the voice, and light of the external environment would lead to the change of brain activity, so we should strictly control each step of the experimental preparation, such as audio-visual closed, rest before the test, and acupuncture preparation. As far as possible, we ought to unify the time of acupuncture therapy and PET/PET-CT scans.

## 5. Conclusion

This paper focuses on the scanning process and experimental quality control to analyze the methodological issues involved in the acupuncture-PET/PET-CT studies in detail. The results provide useful references for future PET/PET-CT study and even PET-MR study.
